# Preterm Neonatal Mortality and its predictors in Tikur Anbessa Specialized Hospital, Addis Ababa, Ethiopia: a retrospective cohort study

**DOI:** 10.4314/ejhs.v31i1.6

**Published:** 2021-01

**Authors:** Yared Asmare Aynalem, Hussien Mekonen, Tadesse Yirga Akalu, Bereket Gebremichael, Wondimeneh Shibabaw Shiferaw

**Affiliations:** 1 College of Health Science, Debre Birhan University, Debre Birhan, Ethiopia; 2 College of Health Science, Addis Ababa University, Addis Ababa, Ethiopia; 3 College of Health Science, Debre Markos University, Debre Markos, Ethiopia

**Keywords:** Preterm, mortality, incidence, Ethiopia

## Abstract

**Background:**

Preterm neonatal death is a global problem. In Ethiopia, it is still high, and the trend in reduction is slower as compared to child mortality. Preterm neonatal birth is the leading cause. The magnitude and associated factors are also not well documented. Therefore, this study aimed to estimate the incidence of mortality and its predictors among preterm neonates in Tikur Anbesa Specialized Hospital (TASH).

**Methods:**

An institution-based retrospective cohort study was conducted among 604 preterm neonates admitted to Tikur Anbesa Specialized Hospital. Data were collected by reviewing patient charts using systematic sampling with a checklist. The data entry was done using EpiData version 4.2, and analysis was done using Stata Version 14.1. Kaplan-Meier and log-rank tests were used to estimate the survival time and to compare it. Cox proportional hazard was also fitted to identify major predictors. Hazard Ratios (HRs) with 95% Confidence Intervals (CI) were used to assess the relationship between factors associated with the occurrence of death. Finally, statistical significance was declared at p-value < 0.05.

**Results:**

In this study, a total of 604 patient charts were reviewed; of these, 571 met the inclusion criteria and were recruited to the study. A total of 170(29.7%) preterm neonates died during the follow-up period. The median follow-up time of preterm neonate under the cohort was 21 days (IQR: 4, 27). The incidence rate was 39.1 per 1000-person day. Rural residency (AHR: 1.45 (95% CI: 1.1,4.8)), Maternal diabetic Mellitus (AHR:2.29 (95%CI: 1.43,3.65), neonatal sepsis (AHR:1.62 (95% CI: 1.11,2.37), respiratory distress (AHR:1.54 (95% CI:1.03, 2.31), extreme prematurity (AHR:2.87 (95% CI:1.61, 5.11), and low APGAR score (AHR:3.11 (95% CI:1.79, 5.05) was found to be predictors.

**Conclusion:**

The rate of preterm neonatal mortality is still an important problem. Having maternal gestational Diabetic Mellitus, neonatal sepsis, respiratory distress, and low Apgar score were major predictors for preterm neonatal mortality. Therefore, efforts have to be made to reduce the incidence of death and for timely management of mothers with Diabetic Mellitus. Healthcare professionals should also work on early diagnosis and treatment of preterm neonate with sepsis, respiratory distress, and low Apgar score.

## Introduction

A preterm neonate is defined as a baby born alive before 37 completed weeks of pregnancy from the first day of the last menstrual period (1). The death of preterm newborns with in the first 28 days of neonatal life describes preterm neonatal mortality. The survival of these neonates has improved significantly through improved and specialized NICU care. Nevertheless, it remains the main reason for neonatal admission, death, and risk of lifelong sequel (2). In 2016, more than 15 million babies were born as preterm newborn. From these estimates, 60%–85% were in Africa and South Asia (3). This figure indicates that even if preterm neonatal death is a global public health problem, it is more prevalent in developing countries (4). As a result, it is a serious issue in developing nations (1–7). It is also the first leading cause of neonatal mortality followed by prenatal asphyxia and neonatal sepsis (8).

In developed countries, 50% and 90% of preterm babies born at 24 and 28 weeks of gestation survived, but less than 10% of these babies survived in low-income nations (9). Globally, there are different policies, strategies, and programs that work towards prevention and care of preterm birth and reducing mortality (5,6). Yet, still it is the first leading cause of neonatal mrtality and the second most common cause of under-five mortality (6–8). Sixty percent of underfive child death also occurred in the neonatal period in which preterm birth and its complications contributed to 35% of it (9,10).

In Ethiopia, the report of the Ethiopian Demographic and Health Survey (EDHS) indicated that 30 per 1000 live births neonatal mortality again is primarily related to prematurity (10). The proportion of preterm neonatal death in Ethiopia was also reported to be 25.1–32.9% in Gondar, 34.9% in Jimma (11), and 29% in a study done at five selected hospitals (Gondar University Hospital, Jimma University Hospital, Ghandi Memorial Hospital, Tikur Anbessa Hospital and St Paul's Hospital Millennium Medical College) (12)**.** The overall median length of hospital stay for preterm neonates in different studies also varied from 7 days in Amhara to 21 days in the Tigray region (13). Ethiopia developed different policies and programs including expanding Neonatal Intensive Care Unit (NICU), integrated management of neonatal and childhood illness, and quality improvement program to tackle newborn death at institution and community levels by controlling major neonatal complications (14). Besides these efforts, preterm neonatal mortality is still persistently high (11,12,14,15). Additionally, different studies done in Ethiopia determined the prevalence and associated factor of preterm neonatal death. However, limited studies were done on time to death. Furthermore, as far as our knowledge is concerned, there is no research conducted on time to death preterm neonates in the study area. Preterm birth causes most of infant deaths, and it continues to be a significant public health difficulty by increasing the cost of healthcare for developing countries including Ethiopia. These extra expenses might affect both the parents, families as well as the community at large. So, tackling preterm newborn birth and the survival gap might have a double impact to end the preventable deaths of newborns and under-five children. Therefore, this study aimed to determine the preterm neonatal mortality and its predictors among neonates admitted to the NICU of Tikur Anbesa Specialized Hospital. It will also give additional information to planners and programmers to give adequate attention and allocate resources and infrastructures for better care for preterm babies.

## Methods

**Study design, setting, and population**: An institution-based retrospective cohort study was conducted among preterm neonates delivered and admitted to Tikur Anbesa Specialized Hospital (TASH) from January 1, 2013, to February 30, 2018. The study was conducted in Addis Ababa, the capital city of Ethiopia at TASH. Addis Ababa has ten sub-cities; the city lies at an altitude of 7,546 feet (2,300 metres). It has twelve governmental and nine nongovernmental hospitals. TASH is one of the governmental hospitals, which has 600 beds in medical, gynecological and obstetrics, surgical, pediatrics, emergency and Outpatient Department (OPD). The NICU of the TASH ward can accommodate a maximum of 60 patients with an average of 20–40 daily admissions. Altogether, 25,000 newborn neonates were admitted to the neonatal intensive care unit in the past five years; of these 5,000 neonates were admitted due to preterm birth. Our source population was all preterm birth neonates admitted at the NICU of TASH (16).

**Study population**: All preterm neonates that were admitted at NICU from the first of January 1, 2013, to February 30, 2018) at TASH

**Sampled population**: All preterm neonates that were admitted at NICU from the first of January 1, 2013, to February 30, 2018) and who fulfilled the inclusion criteria.

**Study unit**: Each selected preterm neonate's chart from the hospital's NICU registry log book

**Eligibility criteria**: All preterm babies' chart in the previous five years (from January 1, 2013, to February 30, 2018) were recruited. However, those with incomplete cards (cards that missed to register at list the following data: date of admission, date of the last contact, status of the neonates and other major predictors) were excluded.

**Sample size determination and sampling procedure**: The sample size was determined using both single population and double population proportion exposure difference formula. The sample size for the proportion of mortality was calculated with the following assumptions: Z α/2; standardized normal distribution value for the 95% CI,1.96, the proportion of mortality rate in a similar study, p = 34.9% (11), d; margin of error taken as 5%, which yields a total of 384. The sample size for predictors was also determined by considering the following assumptions: 95% CI, power 80% the ratio of unexposed to exposed 1:2 and 10% for incomplete records. It was calculated by considering sepsis, jaundice, prenatal asphyxia, ANC, gestational age, weight of neonate and multiple pregnancies (11,17). Moreover, the sample size calculated using gestational age was considered as a final sample size since it gives the maximum size (n=604). Simple random sampling technique was used to recruit a predetermined sample size by using registration number

Dependent variable was incidence of death while independent variables were Neonatal- age at admission, gestational age, sex, the weight of neonate, date of NICU admission, and discharge. Maternal- age, residence

**Gynecologic-obstetric related factors**: having Antenatal Care (ANC) follow-up, gravidity, parity, mode of delivery, multiple pregnancies, PROM, preeclampsia, abruption placenta

**Medical disorders in mothers**: Hypertension, DM, HIV/AIDS, anemia

**Neonatal outcomes**: Apgar score, RD, sepsis, jaundice, hypothermia, PNA, hypoglycemia, meningitis, esophageal atresia.

**The following operational definitions are used in for this study.**

**Censored**: Preterm neonates that were admitted at NICU, but still alive at the end of the study or lost to follow-up including discharged to home, discharged against medical advice, or transfered out to other health institutions

**Follow-up time**: From the time of admission until either an event or censorship occurs

**Survival status**: The outcome of the premature neonate, either death or censored

**Time origin**: Admission of the preterm neonate at NICU

**Time scale**: Days from the admission of preterm neonates to the last of neonatal period

**Event**: Preterm neonatal mortality

**Data collection method, instruments and quality control**: Before data collection, the chart was evaluated, and charts were identified by their medical registration number. Then, data were retrieved using a pretested checklist which was prepared in English from HMIS registration format and patient's card. Trained nurses collected the data. The starting point for the follow-up was the time from first date of NICU admission to the date of death, the date of death, censored, or end of study (until February 30, 2018). Death was confirmed by reviewing the medical death certificate in the hospital. The checklist was also evaluated by experienced researchers. Language clarity, appropriateness of data collection tools, estimated time to completion, and the necessary amendments were considered based on the pretest. Intensive training was given concerning the data abstraction tool and data collection process for both data collectors and supervisors. During the data collection time, close supervision and monitoring were done.

**Data processing, analysis, and presentation**: Before analysis, data were cleaned, edited, and coded. Any errors identified at this time were corrected after a review of the original data using the code numbers. Data was entered using Epi-Data version 4.2 and analyzed using STATA 14 statistical software. The Incidence Density Rate (IDR) was calculated for the entire study period. Subsequently, the number of mortalities within the follow-up period was divided by the total person-time at risk on follow-up and reported per 1000-person days. The Kaplan-Meier curve was used to estimate mean survival time, and log-rank tests were used to compare survival curves. Before running the Cox proportional hazards regression model, multi-collinearity was checked. The Cox-proportional hazard regression model assumption was also checked using Schoenfeld's residual test with variables having p-values greater than 0.05 being considered as fulfilling the assumption. The bivariable Cox-proportional hazards regression model was fitted. Hazard Ratios (HR) with 95% Confidence Intervals (CI) were used to assess the relationship between factors associated with the occurrence of death. Finally, statistical significance was declared at p-value < 0.05.

**Ethical consideration**: A written letter of permission from the Research Committee, Addis Ababa University, College of Health Science, was obtained and submitted to TASH. Oral permission was obtained from TASH coordinators. Confidentiality of the patient profiles was ensured throughout the research process.

## Results

**Socio-demographic characteristics of the study participants**: Among 604 preterm neonate charts reviewed, 571(94.54%) met the enrollment criteria in the final analysis, and 33 charts were excluded (10 unrecorded dates of admission, 10 unrecorded dates for last visits, 7 of the chart were not available at the time of data collection and 6 of them did not have a written death certificate). Among 571 study participants, almost half (52.4%) were males and near to two-third of their mothers, 382(66.90 %), came from urban areas. Neonates in the early neonatal period accounted for 275(48.2%) of the study participants. The majority of the mothers, 426(74.61%), belonged to the age group of 20–35 (Table 1).

**Table 1:** Baseline socio-demographic characteristics of preterm neonates and their mothers in TASH, Addis Ababa, Ethiopia, from January 1, 2013, to February 30, 2018

Covariates	Category	Frequency	Percentage
Sex	Female	272	47.6
	Male	299	52.4
Neonatal age	<24 hrs.	275	48.2
	1–7 day	262	18245.9
	>7 day	34	255.95%
Mater age	<20	61	10.9
	20–34	426	74.6
	>34	84	14.7
Residency	Ruler	188	32.9
	Urban	383	67.1
GA	<28	31	5.4
	28–32	208	50.6
	32–37	332	58.1
weight of	<1000	33	5.8
Newborn	1000–1500	155	27.1
	15000–2500	341	59.7
	>2500	42	(7.4)

**Maternal and pregnancy characteristics**: Among the total mothers enrolled in the study, 75(30.65%) had preeclampsia, 165(28.9%) had PROM, 76(13.3%) had HIV/ADIS, and 56(9.8%) had DM. The results of this study also indicated that the majority, 410(84.5%), of the mothers had a history of ANC visits (Table 2).

**Table 2:** Maternal and pregnancy characteristics of the study participant that was admitted to the NICU of TASH, Addis Ababa, Ethiopia, from January 1, 2013, to February 30, 2018

Characteristics	Category	Frequency	Percentage
Preeclampsia	Yes	175	30.6
	No	396	69.3
PROM	Yes	165	28.9
	No	406	(71.1)
Abruption	Yes	18	3.15
Placenta	No	553	96.8
ANC follow up	Yes	481	84.2
	No	90	15.8
Number	<2	439	76.9
Parity	>2	132	23.1
HIV/ADIS	Yes	76	13.3
	No	495	86.7
DM	Yes	56	9.8
	No	515	90.2

**Figure 1:**
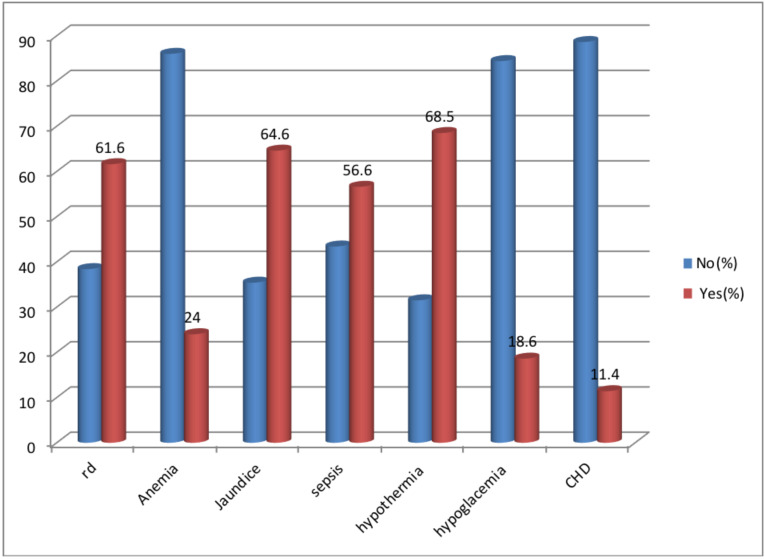
Commonly reported causes of death for preterm neonates that were admitted to NICU of TASH, Addis Ababa, Ethiopia, from January 1, 2013, to February 30, 2018

**Commonly reported neonatal related causes of death for preterm**: Commonly reported causes of death for preterm neonates were hypothermia, 391(68.5%), RD, 352 (61.65%), and neonatal Sepsis, 323 (56. 57%). The other common causes were jaundice, hypoglycemia, and anemia (Figure 1).

**Incidence of death among preterm neonates**: The preterm neonates, who were admitted to NICU, were followed from 0 to 28 days. In the current study, 170(29.78%) (95% CI: 23–33) of the study participants died. The overall incidence rate of preterm neonate mortality was 39.1 deaths per 1000 person-days (95% CI: 33.59, 45.38) which gave a total of 4354 neonate-days observation follow-up time.

**Figure 2:**
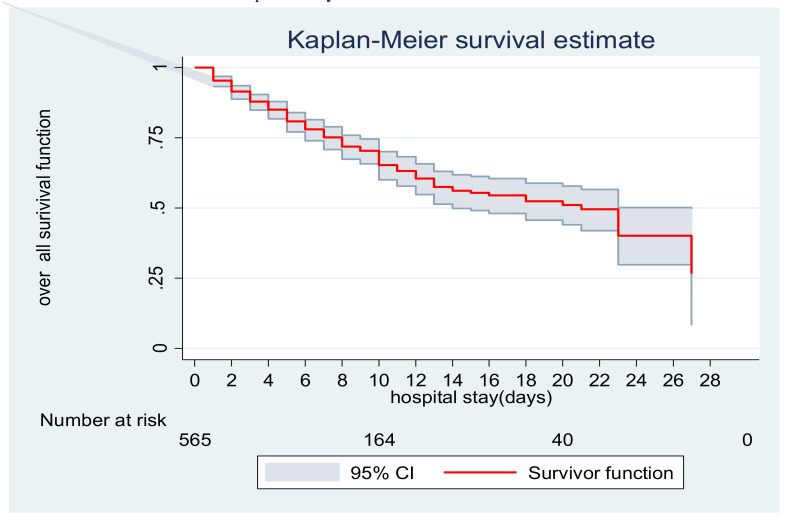
Overall Kaplan-Meier survival estimate of preterm neonates admitted to the NICU of TASH, Addis Ababa, Ethiopia, from January 1, 2013, to February 30, 2018.

**Overall survival function**: The overall Kaplan-Meier estimate showed that the probability of survival of preterm neonates was higher on the first day of admission, and increased failure to survive throughout the follow-up period (Figure 1). During the first day of the hospital stay, the maximum probability of survival (95.4%) was observed with SE±0.01 (95% CI: 0.93, 0.97). The overall median survival time was found to be 21 days with an interquartile range of (4,27) (Figure 2).

**Figure 3:**
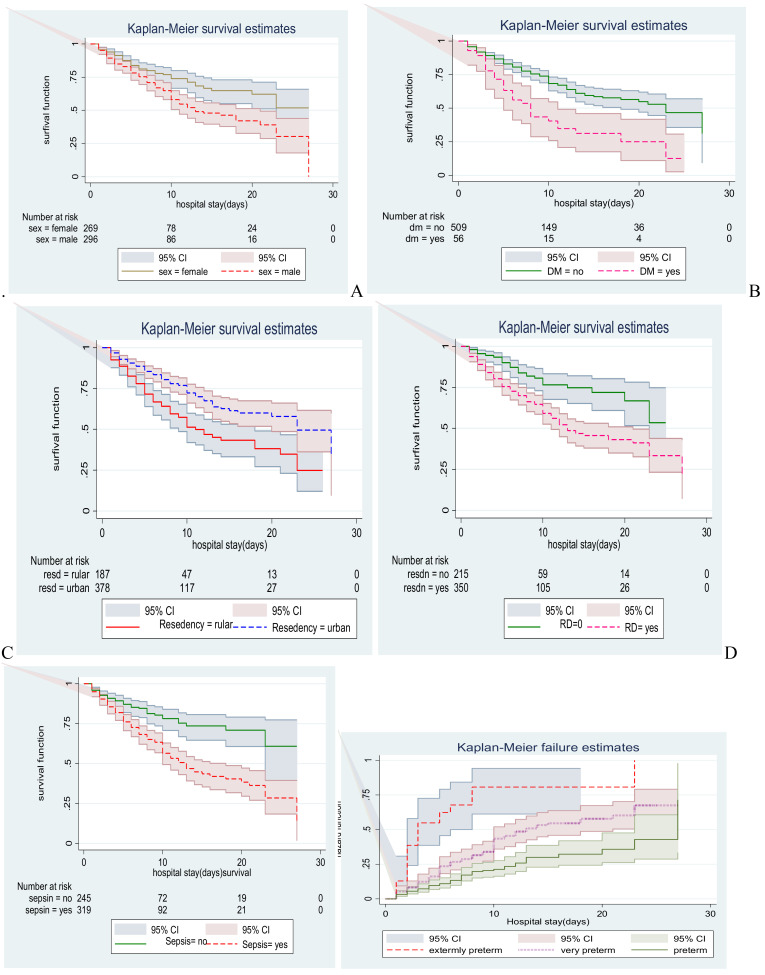
Kaplan-Meier survival curve of preterm neonates admitted in NICU by A: sex, B: gestational DM.C: residency, D: RD, E: sepsis, F: gestational age, Addis Ababa, Ethiopia, from January 1, 2013, to February 30, 2018 N.B. The observed difference seen in the plot was checked using the Cochran-Mantel Haenszel log

**Comparison of Survivorship Functions for different categorical variables**: In this study, male neonates had lower survival time compared to females. At the 27^th^ day of hospital stay, the overall survivals of males and females were found to be 52% and 30% respectively. The current study also revealed that neonates born from mothers who were non-diabetic at baseline of admission had a longer survival time than those born from mothers with DM. In this study, neonates without sepsis had more favorable survival probability than neonates with sepsis (Figure 3).

**Model comparison criteria**: The goodness of model fitness was checked using the Cox-Snell residual test. Based on the Akaike Information Criterion, the univariate semi-parametric Cox-proportional (AIC = 435.8) model was more efficient than parametric exponential (AIC = 987.5) and Weibull (AIC = 686.9) hazard Gompertz hazard distribution (AIC =1123.54) model.

**Fitted Cox proportional hazard model for predictors of preterm neonatal mortality**: The result of the multivariable analysis showed that preterm neonates whose mothers came from rural locations were 1.45 times higher risk to die as compared to those from urban areas (AHR: 1.45 (95% CI: 1.1,4.8)). The hazard ratio for preterm neonates who were born to mothers with DM was also 2.3 times more likely to die as compared to those neonates born from mothers without DM (AHR:2.29 (95% CI:1.43,3.65)). Preterm neonates with RD at the baseline were also 1.5 times more likely to die (AHR: 1.5 (95%CI: 1.03, 2.31). The HR for death was 1.51 times (AHR: 1.51, 95%CI: 1.16, 2.13) higher in male patients (Table 4).

**Table 4:** Results of the bivariate and multivariate Cox regression analysis of preterm neonate that were admitted to NICU, TASH, Addis Ababa, Ethiopia, from January 1, 2013, to February 30, 2018

Predictor	Category	Death	Censored	Total	CHR (95% CI)	AHR (95% CI
		(%)	(%)	(%)		
Sex	Female	60(35.3)	212(52.9)	272(47.6)	1	
	Male	110(64.7)	189 (47.1)	299(52.4)	1.70(1.24,2.33)**	1.51(1.06,2.13)*
Residency	Rural	92(54.1)	111(27.7)	188(32.9)	1.92(1.4–6.7) **	1.45(1.1,4.8) *
	Urban	78(45.9)	291(72.3)	383(67.1)	1	
PROM	Yes	58(34.1)	107(26.7)	165(28.9)	1.48(1.08, 2.04)*	1.07(0.76,1.52)
	No	112(65.9)	294(73.3)	406(71.1)	1	
Preeclampsia	Yes	72(42.3)	103(25.7)	175(30.6)	1.61(1.18, 2.18)**	0.88(0.61,1.25)
	No	98(57.7)	298(74.3)	396(69.3)	1	
Maternal DM	Yes	34(20)	22(5.5)	56(9.8)	2.38(1.63,3.46)***	2.29(1.43,3.65)***
	No	136(80)	379 (94.5)	515(90.2)	1	
RD	Yes	219(38.3)	219 (38.3)	379(94.5)	2.27(1.56 ,3.28)***	1.54(1.03, 2.31)**
	No	219(38.3)	219 (38.3)	219 (38.3)	1	
Sepsis	Yes	139(81.8)	252(62.8)	323 (56.6)	2.21(1.57,3.12)***	1.62(1.10, 2.37)**
	No	44(25.9)	149(37.2)	247(43.3)		
Gestational	26–28	23(13.5)	8	31(5.4)	6.31(3.89,10.24)***	2.87(1.61,5.11)***
Age	28–32	86(50.6)	122(30.4)	208(36.4)	1.96(1.41,2.72)***	0.95(0.64,1.41)
	32–37	61(35.9)	271(67.6)	332(58.1)	1	
First minute	<7	154(90.6)	283 (70.5)	437 (76.5)	3.22(1.92,5.39)*	3.11 (1.79,5.05)*
APGAR Score	≥7	16	118 (29.5)	134 (23.5)	1	
Fifth minute	<7	128(75.2)	131(32.7)	259(45.4)	3.83(2.70, 5.44)***	1.81(1.32,4.78)***
APGAR score	≥7	42(24.8)	270(67.3)	312 (54.6)	1	

NB: * Significant (P < 0.05), ** (p<0.01), *** (p<0.001)

## Discussion

In this retrospective cohort study, we aimed to determine the preterm neonatal mortality and its predictors that were admitted to NICU at TASH. According to the current finding, the proportion of neonatal mortality was 29.7%. This finding is lower than the finding of a study conducted in Jimma (34.9%) (11). However, it is higher than a study conducted in Arba Minch 19.4% (18). Moreover, it was consistent with other previous studies conducted in Ethiopia (25.2%) (17) and other African countries like Nigeria (27.69% (15,17) and Johannesburg (26.5%) (19). This variation may reflect the difference in sample size, methodology, or study periods, which may reflect changes in treatment modalities, which are also potential explanations. This difference might also be due difference in study area, in which the current study includes only preterm neonates' most the riskiest group.

overall incidence of mortality was 39.1 deaths per 1000 person-day observation, which exceeds both reports by EDHS 2019 30% per 1000 (10), and China (20). However, it is lower than a study in Jordan (21). In contrast, it is higher than a study conducted in Tigray (13). This marked difference might be attributed to several factors such as care difference in which developed countries might be better equipped with skilled professionals, support personnel, and equipment to perform neonatal resuscitation, evaluate and provide postnatal care of a newborn. The other possible justification might be the characteristics of the study participants. For example, a study in the Tigray region includes both premature and mature neonates in the study.

The risk of mortality was higher in the early neonatal period with an incidence rate of 40.1/1000 live birth, which is similar to a study in Jordan (21). The possible explanation might be related to pregnancy-related complications and/or birth resulting in delays in identification and poor management by health workers.

This study also showed that male neonates are nearly twice as likely as females to die (48.9 vs 28.5 deaths per 1,000 live births). This result is in line with EDHS 2016 (22). The reason for this could be hormonal environment differences which might be associated with differences in pulmonary biomechanics and vascular development that lead to increased respiratory and neurological morbidity among premature males (15).

The overall mean and median survival time were 17 and 21 days respectively, consistent with studies in Ethiopia; UOGH (17) and Jimma University Hospital (11)). The survival curves in our study were significantly different between neonates with sepsis and without sepsis. The consistent result was reported in Jimma (11). In this study, the risk of preterm neonatal death among cases of sepsis was nearly two times higher than non-cases. This finding is supported by a result of studies conducted in developed and developing country (11, 17, 23).

Being a single tone pregnancy was higher hazard death as compared to multiple pregnancies which; this is consistent with different studies (17,24,25). This might be due to the stressor effect of multiple gestations for the fetus, which subsequently may lead to premature death. In our study, the incidence of death in males was two times higher than female neonates. This result is in agreement with other studies (11,17,26). This finding may reflect the delay of lung maturation among male premature neonates. Preterm neonate diagnosis with RD had 1.5 times risk of death than its counterpart. Consistent results have been recorded in our country and in studies from other countries (17,18). This may be due to lung immaturity and maternal factor like having DM, PROM which may increase alveolar surface tension (4).

This finding showed that neonates came from the rural area were 1.4 one point four times more likely today than its counterpart. This finding was supported with previous studies (34.9%) (11, 17). This difference could be due to rural residents remaining relatively disadvantaged in terms of infrastructure, knowledge and awareness, distance from services, and socioeconomic differences. The current finding showed that neonates delivered from mothers who had DM have increased risk of death by two folds as compared to neonates from none DM mothers. This finding is comparable to a study done at UOGH %) (17). It could be that preterm neonates born to DM women may have abundant glucose stores but develop hypoglycemia because of hyperinsulinemia induced by maternal and fetal hyperglycemia. Neonates whose first and fifth-minute APGAR scores of less than 7 were two and three times more likely to die. This study is supported by studies previously done in Ethiopia (11,17,18). This might be because of delay in identification of newborn complications and its mismanagement and misdiagnosis.

In conclusion, in this study, the proportion of preterm neonatal death was high compared to findings of studies in Ethiopia and other countries. Cox proportional hazard analysis showed that the major factors of preterm neonatal mortality were being male, living in rural area, having maternal DM, neonatal sepsis, RD, GA less than 28 weeks, and low APGAR score. Therefore, TASH should be able to strengthen careful follow-up and regular monitoring of preterm neonates. Special emphasis and close follow-up should also be given to male neonates, rural residency, and have low APGAR score, sepsis, and RD. It would be better to strengthen the screening of DM during pregnancy and give priorities to premature neonates born from DM mothers. A longitudinal prospective cohort study is strongly encouraged to identify the long-term outcomes of preterm newborn births, and the health needs of babies who survive as prematurity and to identify other predictors including socioeconomic, genetic, and environmental and other factors as well as reason specific predictors.

## Figures and Tables

**Table 3:** Mean and median survival time and log-rank test for equality of survivor functions for preterm neonates that were admitted to NICU of TASH, Addis Ababa, Ethiopia, from January 1, 2013, to February 30, 2018

Variables	Category	Survival		Log-rank test (x^2^)

		Median	Mean (95% CI)	
Mother age	<20	16(7,22)	14(11.3, 16.8)	10.3
	20–34	23(20.1,35)	18.5(11.3, 16.8)	
	>34	11(9.9,23)	14.0(11.6, 16.5)	
Delivery	Cesarean	14 (12,16.2)	16.2(14.5, 17.9)	4.33
	Spontaneous	23(20,24.5)	18.4(16.8, 19.9)	
	Instrumental	10(1,13.1)	11.1(5.6, 16.6)	
Preeclampsia	Yes	13(10,23)	15.0(13.1, 16.9)	9.86**
	No	23 (18,26.1)	18.3(16.9, 19.8)	
DM	Yes	8(5,11)	11.1(8.5, 13.6)	22.49***
	No	23(18,24.1)	18.2(16.9, 19.4)	
Jaundice	Yes	13(10,23)	15.4(13.6,17.2)	5.83**
	No	23(21,24.8)	18.5(17.1,19.9)	
Sepsis	Yes	13(11,18)	14.9(13.54,16.41)	22.84***
	No	16(13.2,18.3)	20.8(19.2,22.6)	
Hypoglycemia	Yes	12(10,23)	13.7(11.7, 15.8)	9.17***
	No	21(20,26.9)	18.2(16.9,19.6)	
GA	<28	3(2,6)	7.3(4.1,10.6)	71.08***
	32–37	27(23, 27.3)	20.2(18.7,21.8)	
Wight	<1000	5(3, 13)	9.92 (6.5,13.3)	
	1000–1500	23(12,25.4)	17.1(15.1,19)	24.34*
	1500–2500	23(16,24.1)	17.1(15.1,18.4)	
	≥2500	20(10,21.6)	18.6(13.8,23.5)	
ANC follow up	Yes	23(21,23.5)	18.8(17.6, 20.0)	36.8**
	No	10(6,10)	10.4(8.5, 12.3)	
Multiple pregnancies	Yes	13(10,23)	14.5(12.9, 16.1)	8.84***
	No	27(20,29.2)	18.8(17.4, 20.3)	
Breastfeed	Yes	27(22.3,28.7)	20.5(19.2, 21.8)	80.18***
	No	8 (7, 10)	10.7(9.2, 12.2)	
5^th^ minute APGAR	<7	10 (9 ,12)	12.8(11.3,14.2)	68.6***
	≥7	27(27, 28)	22.3(16.1, 18.4)	
